# Anode-assisted electro-fermentation with *Bacillus subtilis* under oxygen-limited conditions

**DOI:** 10.1186/s13068-022-02253-4

**Published:** 2023-01-10

**Authors:** Yu Sun, Marika Kokko, Igor Vassilev

**Affiliations:** Faculty of Engineering and Natural Sciences, Tampere University, Korkeakoulunkatu 8, 33720 Tampere, Finland

**Keywords:** *Bacillus subtilis*, Oxygen supply, Anode-assisted electro-fermentation, Anode respiration, Acetoin bioproduction

## Abstract

**Background:**

*Bacillus subtilis* is generally regarded as a ubiquitous facultative anaerobe. Oxygen is the major electron acceptor of *B. subtilis*, and when oxygen is absent, *B. subtilis* can donate electrons to nitrate or perform fermentation. An anode electrode can also be used by microorganisms as the electron sink in systems called anodic electro-fermentation. The facultative anaerobic character of *B. subtilis* makes it an excellent candidate to explore with different electron acceptors, such as an anode. This study aimed to optimise industrial aerobic bioprocesses using alternative electron acceptors. In particular, different end product spectrum of *B. subtilis* with various electron acceptors, including anode from the electro-fermentation system, was investigated.

**Results:**

*B. subtilis* was grown using three electron acceptors, i.e. oxygen, nitrate and anode (poised at a potential of 0.7 V vs. standard hydrogen electrode). The results showed oxygen had a crucial role for cells to remain metabolically active. When nitrate or anode was applied as the sole electron acceptor anaerobically, immediate cell lysis and limited glucose consumption were observed. In anode-assisted electro-fermentation with a limited aeration rate, acetoin, as the main end product showed the highest yield of 0.78 ± 0.04 mol_product_/mol_glucose_, two-fold higher than without poised potential (0.39 ± 0.08 mol_product_/mol_glucose_).

**Conclusions:**

Oxygen controls *B. subtilis* biomass growth, alternative electron acceptors utilisation and metabolites formation. Limited oxygen/air supply enabled the bacteria to donate excess electrons to nitrate or anode, leading to steered product spectrum. The anode-assisted electro-fermentation showed its potential to boost acetoin production for future industrial biotechnology applications.

**Supplementary Information:**

The online version contains supplementary material available at 10.1186/s13068-022-02253-4.

## Background

In industrial biotechnology, oxygen is essential throughout the entire aerobic process to promote rapid cell growth and support microorganisms to maintain their metabolic functions and balance their metabolic redox state [1, 2]. The bioprocesses with high oxygen demand often face issues, such as high operational expenses, poor liquid–gas mass transfer rates and fast foam generation [3–6]. In many oxygen-dependent systems, the overall yields are limited by unsolicited cell growth and unbalanced metabolisms. The strategies used to improve oxygen independence in aerobic processes include separating the cell growth and product formation into multiple steps, utilising synthetic biology and metabolic engineering tools, or substituting oxygen with alternative electron acceptors [1, 2]. However, many current practices are limited to certain anaerobic or facultative anaerobic microorganisms [7–9], and future studies should focus on how to demote the oxygen dependency of industrially relevant microorganisms during the aerobic process.

Anaerobic processes include microorganisms performing fermentation or anaerobic respiration. In fermentation, fermentable substrates, like polysaccharides or sugars, are converted into pyruvate and then further oxidised into organic acids and alcohols [10]. In anaerobic respiration, some compounds, such as nitrate, sulphate, fumarate, iron (III), manganese (IV) or CO_2_ are used as the terminal electron acceptor [11]. Anaerobic processes are often preferred especially in industrial applications due to their low costs and relatively high volumetric production rates. However, compared to aerated systems, anaerobic respiration and fermentation come with less energy conservation, undesired by-product formation and requirement for complex downstream processes [2, 12].

Facultative anaerobic bacteria, including *Escherichia coli*, *Lactobacillus sp.* and *Bacillus subtilis,* are microorganisms that metabolise energy both aerobically and anaerobically when adapting to oxygen fluctuations [1]. *B*. *subtilis* is a rod-shaped, gram-positive bacterium that can grow aerobically and anaerobically by donating electrons to various terminal electron acceptors [13–16]. Under oxygen-limited conditions, *B. subtilis* is reported to perform anaerobic respiration by nitrate ammonification or mixed acid fermentation where pyruvate is converted into various metabolites, such as lactate, acetate, acetoin and 2,3-butanediol [13, 16].

*B. subtilis* is a well-known cell factory that produces enzymes, heterologous proteins, food additives, vitamins, antibiotics, amino acids and insecticides [17–19]. Two fine chemicals naturally formed by *B. subtilis*, acetoin (3-hydroxybutanone or acetyl methyl carbinol) and its reduced form, 2,3-butanediol, have shown growing market share in food, cosmetics and chemical and agriculture industries during the last decades [20]. At the industrial manufacturing level, bulk production of acetoin and 2,3-butanediol relies on chemical synthesis using fossil feedstocks [20]. Due to the increasing environmental and financial concerns, biotechnological acetoin and 2,3-butanediol production from biomass origin with microorganisms are explored. Most of the acetoin and 2,3-butanediol producer, nonetheless, are pathogenic and cannot be used for industrial starter cultures, e.g. *Serratia, Klebsiella, Enterobacter, Raoultella,* and *Salmonella* [21–23]. *B. subtilis*, on the other hand*,* is recognised as safe by U.S. Food and Drug Administration (FDA) and European Food Safety Authority (EFSA).

The acetoin and 2,3-butanediol production in *B. subtilis* typically use oxygen as the electron acceptor and small molecule sugars (e.g. glucose, xylose or sucrose) as substrate, while the formation of by-products (e.g. lactate, acetate, and ethanol) has been observed. During the acetoin production processes, oxygen can be one of the limiting factors since high dissolved oxygen concentrations are required to re-oxidise NAD(P)H or FADH_2_ and to generate ATP effectively [24]. One novel approach suggested to demote oxygen dependency and steer the metabolism of *B. subtilis* is the use of anodic electro-fermentation [2]. In anodic electro-fermentation, an anode of a bioelectrochemical system (BES) working as an electron sink accepts surplus electrons from microorganisms upon substrate oxidation, which can support a more balanced redox state with fewer undesired by-products [25, 26]. *B. subtilis* was proposed as an ideal starter culture for anodic electro-fermentation due to its ability to produce naturally a redox-active mediator and form a conductive biofilm [2, 27]. Yet, the comprehensive information about *B. subtilis* fermentative process using an anode as an electron acceptor, especially under oxygen-limited conditions, has not been fully revealed.

This study investigated the potential of utilising *B. subtilis* for acetoin and 2,3-butanediol production from glucose under different oxygen supplies and alternative electron acceptors. Three electron acceptors (with different states of matter), i.e. oxygen (gas), nitrate (liquid) and anode from BES (solid), were tested. Furthermore, the effects of anaerobic and oxygen-limited conditions on the use of two additional electron acceptors, i.e. nitrate and the anode, were examined by monitoring the glucose consumption, cell densities and end product formation.

## Materials and methods

### Microbial strain and aerobic cultivation conditions

*B. subtilis* 168 trpC xyl^+^ previously constructed by Averesch & Rothschild [28] for para-aminobenzoic acid production was cultivated in M9 minimal medium amended with 27.8 mM (5 g/L) glucose as the sole carbon source in all experiments. For detailed medium composition, the reader is referred to Additional file 1. The pH of the growth medium was adjusted to 7.1 using 1 M HCl. Agar plates were prepared with the same M9 minimal medium and 15 g/L agar–agar. Pre-cultures were prepared by picking and transferring single colonies from agar plates into baffled shake flasks and placed on a rotary shaker (IKA KS4000i Control, Germany) for aerobic overnight cultivation at 300 rpm and 35 °C. Cells were harvested by centrifugation (at 8000 rpm, 4 °C, 10 min), washed and resuspended in fresh M9 medium before transferring into the main cultivation vessels. Inoculum was added to reach an initial optical density (OD) of 0.1 at 600 nm in shake flasks, and for oxygen-limited experiments higher initial OD (0.4–0.7) was aimed at to ensure enough cells as biocatalysts under limited growth conditions (Table 1). Aerobic experiments were accomplished by using 100 mL shake flasks with 20% liquid volume at 300 rpm and 35 °C. In selected experiments, the real-time cell densities for different concentrations of K_3_[Fe(CN)_6_] (0, 0.5, 1.5 and 5 mM) were monitored using a cell growth quantifier (SBI, Germany).

**Table 1:** Different cultivation vessels and operational parameters tested in this study

Brief name	Condition tested	Gas supplied	Liquid volume	Initial OD (600 nm)	Run time	Addition of 1.5 mM K_3_Fe(CN)_6_
Shake flask	Aerobic	O_2_, freely diffused	20 mL	0.1	12 h	No
Serum flask	Anaerobic amended with 23.5 mM sodium nitrate	N_2_ headspace, 1.4 atm	96 mL	0.5	170 h	No
Serum flask [+ 4% O_2_]	Oxygen-limited amended with 23.5 mM sodium nitrate and 4% O_2_	N_2_ + O_2_ headspace, 1 atm	96 mL	0.4	170 h	No
Anaerobic AP	Anaerobic BES, + 0.7 V	N_2_ continuously purged headspace	300 mL (anolyte)	0.7	170 h	Yes
Anaerobic OC	Anaerobic BES, open-circuit control	N_2_ continuously purged headspace	300 mL (anolyte)	0.7	170 h	Yes
Moderate AP	Oxygen-limited BES, aerated at 30 mL/min + 0.7 V	O_2_, continuously purged medium	300 mL (anolyte)	0.5	66–75 h	Yes
Moderate OC	Oxygen-limited open-circuit control, aerated at 30 mL/min	O_2_, continuously purged medium	300 mL (anolyte)	0.4	66–75 h	Yes
Limited AP	Oxygen-limited BES aerated at 5 mL/min, + 0.7 V	O_2_, continuously purged medium	300 mL (anolyte)	0.6	170 h	Yes
Limited OC	Oxygen-limited open-circuit control, aerated at 5 mL/min	O_2_, continuously purged medium	300 mL (anolyte)	0.6	170 h	Yes
Limited AP [-]	Oxygen-limited BES aerated at 5 mL/min, + 0.7 V	O_2_, continuously purged medium	300 mL (anolyte)	0.6	170 h	No
Limited OC [-]	Oxygen-limited open-circuit control, aerated at 5 mL/min	O_2_, continuously purged medium	300 mL (anolyte)	0.6	170 h	No

### Anaerobic and microaerobic serum flasks

Anaerobic and microaerobic cultivations with nitrate as electron acceptor were done by using 120 mL serum flasks with 80% liquid volume and incubated at 150 rpm and 35 °C. Serum flasks were prepared by initially adding the buffer solution and 23.5 mM (2 g/L) sodium nitrate sparged with N_2_ gas (Woikoski Oy, Finland) for 30 min to remove the dissolved oxygen. Before autoclaving, the headspace was filled with N_2_ at 1.4 atmospheres monitored by a gas meter (LEO1, KELLER, Switzerland). Concentrated glucose and trace element solutions were prepared in separate sterile and anaerobic serum flasks using the same technique. All needles and syringes used for serum flask experiments were flushed with sterile N_2_ gas before adding the concentrated solutions to reach the concentrations of the M9 medium. For microaerobic experiments, the headspace overpressure was released immediately after the inoculum was added and replaced with 4 mL sterile O_2_ gas (Woikoski Oy, Finland).

### Bioelectrochemical system and operation

Bioelectrochemical experiments were prepared using 300 mL H-type borosilicate reactors (Adams and Chittenden Scientific Glass, USA). Detailed BES reactor setup and medium compositions are shown in Additional file 1. K_3_[Fe(CN)_6_] (1.5 mM) was added to the anolytes of selected runs to act as a redox mediator. A circular cation exchange membrane (19.6 cm^2^ projected surface area, CMI-7000, Membranes International Inc. USA) was placed between the anodic and the cathodic chambers. BESs used a three-electrode setup. Anode was made of carbon felt (17.9 cm^2^ projected surface area, 1.1 cm thickness, Alfa Aesar, USA) wrapped around a graphite rod (15 cm × 3 mm, Sigma Aldrich, USA). A platinum wire (10 cm × 0.4 mm, Research Material Ltd, UK) was used as the cathode, and an Ag/AgCl 3 M NaCl reference electrode (RE-5B, BASi, USA) was placed in the anode chamber. All potentials were reported with respect to the standard hydrogen electrode (SHE). The working electrode, counter electrode and reference electrode were connected to a multi-channel potentiostat (VMP3, BioLogic, France) to apply the desired anode potential and record the current.

In advance of anaerobic BES inoculation, anolyte was sparged with N_2_ for 30 min at a 1.5 L/min flow rate to remove dissolved oxygen. Afterwards, N_2_ gas was switched to continuously sparge the headspace of the anodic chamber to assure anoxic conditions during the operation. In aerated BES experiments, the air was sparged through a peristaltic tube (L/S 16, Masterflex, USA) to a liquid medium using mass flow controllers (EL-FLOW FG, Bronkhorst, the Netherlands) at 5 mL/min (limited aeration) or 30 mL/min (moderate aeration) flow rate. N_2_ or air inlet was first attached to sterile vent filters (0.2 µm 50 mm Millex-FG PTFE, Merck, USA) and to gas washing bottles filled with sterile MQ water, then connected to the reactors. Table 1 shows the different gases supplied and some key parameters of the different runs. For each condition tested, every data point was averaged from three to four biological replicates and the standard deviations were calculated. Student’s *t*-tests were used to prove the significant difference for BES results (Additional file 1: Table S2).

### Parameters and metabolites analyses

Liquid samples were taken hourly from shake flasks, or daily from serum flasks and anodic chambers of the BESs. For moderate aeration experiments, there was a slight variation in sampling time points reflected in x-axis error bars as different replicates were run at different time periods. pH was measured using a pH meter (3110, WTW, Germany). Cell density was analysed photometrically using a spectrophotometer (UV-1800, Shimadzu, Japan) with absorbance at 600 nm wavelength. Concentrations of glucose, lactate, succinate, acetate, acetoin, 2,3-butanediol and ethanol were determined using high-performance liquid chromatography (HPLC) after filtering through 0.2 µm syringe filters (CHROMAFIL^®^ Xtra PET-45/25, Germany). An HPLC (SIL-20, Shimadzu, Japan) equipped with a refractive index (RI) detector and a sugar column (Rezex^™^ RHM-Monosaccharide H^+^, 300 × 7.8 mm) was used for glucose analysis under the following conditions: column temperature of 40 °C, injection volume of 50 µL, 5 mM sulfuric acid mobile phase at 0.5 mL/min flow rate and 30 min retention time. Concentrations of lactate, succinate, acetate, acetoin, 2,3-butanediol and ethanol were determined using the same HPLC and column under the following conditions: column temperature of 83 °C, injection volume of 100 µL, 10 mM sulfuric acid mobile phase at 0.5 mL/min flow rate and 30 min retention time. Nitrate (NO_3_^−^) and nitrite (NO_2_^−^) concentrations were determined using ion chromatography (ICS-1600, Thermo Fischer Scientific, USA) equipped with an IonPac AG42-SC (4 × 50 mm) guard column, and an IonPac AS4A-SC (4 × 250 mm) analytical anion exchange column under the similar conditions described in Ref. [29]. The gas samples in the headspace (CO_2_, O_2_ and N_2_) were measured by gas chromatography (GC-2014, Shimadzu, Japan) equipped with a thermal conductivity detector (TCD) using the same column and methods as described in Ref. [30].

### Cyclic voltammetry

Cyclic voltammetry was performed before and after the experiments to characterise the mediator and the anode. The anodic chamber of the H-type reactors was filled with a defined M9 minimal medium, while the cathodic chamber contained a phosphate buffer solution. The same three-electrode setup as shown in Additional file 1: Fig. S1 was used. For the CV measurements, current profiles were recorded by scanning the anode potential at a scan rate of 0.1 mV/s within a potential window from 0.2 to 0.9 V vs. SHE, to measure oxidation and reduction reactions of the solution. During the measurements, the temperature was controlled at 35 °C and stirring was switched off. Three cycles (repeats) were recorded at each CV measurement.

## Results

### Aerobic respiration and nitrate respiration of *B. subtilis*

**Fig. 1:**
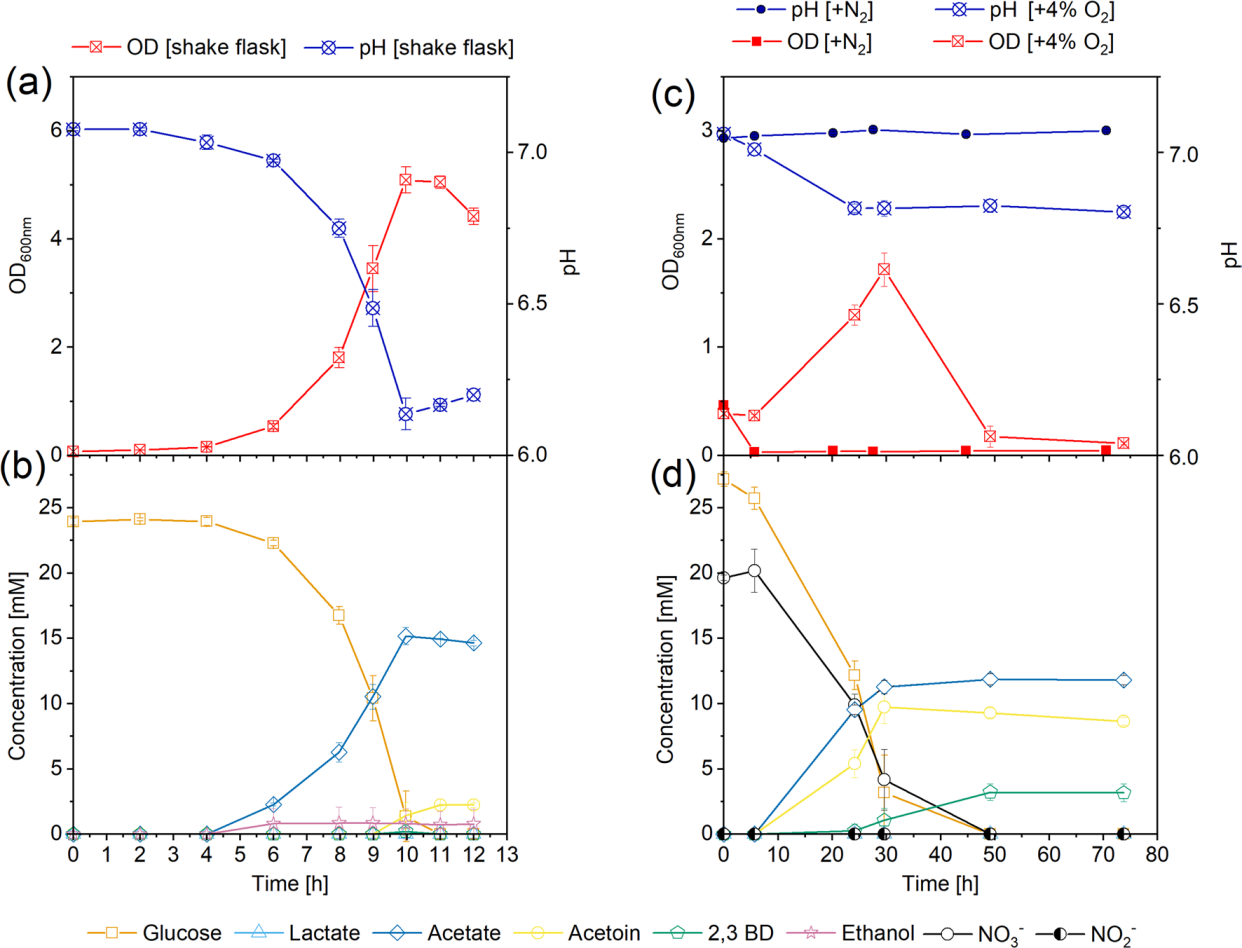
*B. subtilis* grown in aerobic shake flasks: pH and optical density (OD) **a**, glucose consumption and main metabolites measured from aerobic shake flasks **b**, and *B. subtilis* grown in serum flasks amended with 23.5 mM (2 g/L) sodium nitrate under the anaerobic [+ N_2_] and microaerobic conditions [+ 4% O_2_]: pH and OD **c**, glucose, nitrate consumption and end products concentrations measured from the serum bottles under microaerobic condition **d**. All tested reactors and parameters are given in Table 1. Results are based on average data of three to five biological replicates and standard deviations are represented as error bars

Oxygen or nitrite as the electron acceptors for *B. subtilis* was first tested, and cell growth and formation of metabolites were measured. When oxygen was used as the electron acceptor, glucose was rapidly depleted within 10 h with a consumption rate of 2.3 ± 0.1 mmol/L/h, at which time point the maximum cell density (*OD*_600_ = 5.0) was reached and the pH dropped from 7.0 to 6.2 (Fig. 1a, b). Acetate (14.6 ± 0.2 mmol/L) was the main end product besides biomass, along with low titres of acetoin (2.2 ± 0.2 mM) and ethanol (2.0 mM, only measured in one replicate). Overall, aerobic growth gave approximately 61.5% carbon and redox balances (for equations, see Calculations, Additional file 1; for all carbon balances and redox balances calculated, see Additional file 1: Table S1), and acetate presented the highest product yield of 0.7 ± 0.2 mol_product_/mol_glucose_ (for all production rates and yields calculated, see Additional file 1: Table S1).

Nitrate could not be used as the sole electron acceptor under anaerobic conditions. Immediate cell lysis (Fig. 1c) with no substrate consumption nor end products formation was detected in the fermentation broth. Nevertheless, *B. subtilis* performed nitrate respiration under the microaerobic condition when oxygen (4%) was injected into the headspace of the serum flasks. After the headspace oxygen level dropped from 4% to below 1% in 8 h after the inoculation, the nitrate concentration started to decrease and completely consumed after 50 h (Fig. 1d), resulting in a 0.5 ± 0.1 mM nitrate consumption rate. Compared to aerobic growth, lower cell density (*OD*_600_ = 1.7, Fig. 1c) was measured in serum bottles with 0.7 ± 0.2 mM glucose consumption rates. Main metabolites were acetate (11.9 ± 0.4 mM), acetoin (9.7 ± 1.2 mM) and 2,3-butanediol (3.2 ± 0.6 mM). By the end of the experiment, approximately 23 mL CO_2_ was measured from the headspace (Additional file 1: Fig. S2), with 67% carbon and redox balances.

### Anaerobic anode respiration of *B. subtilis*

**Fig. 2:**
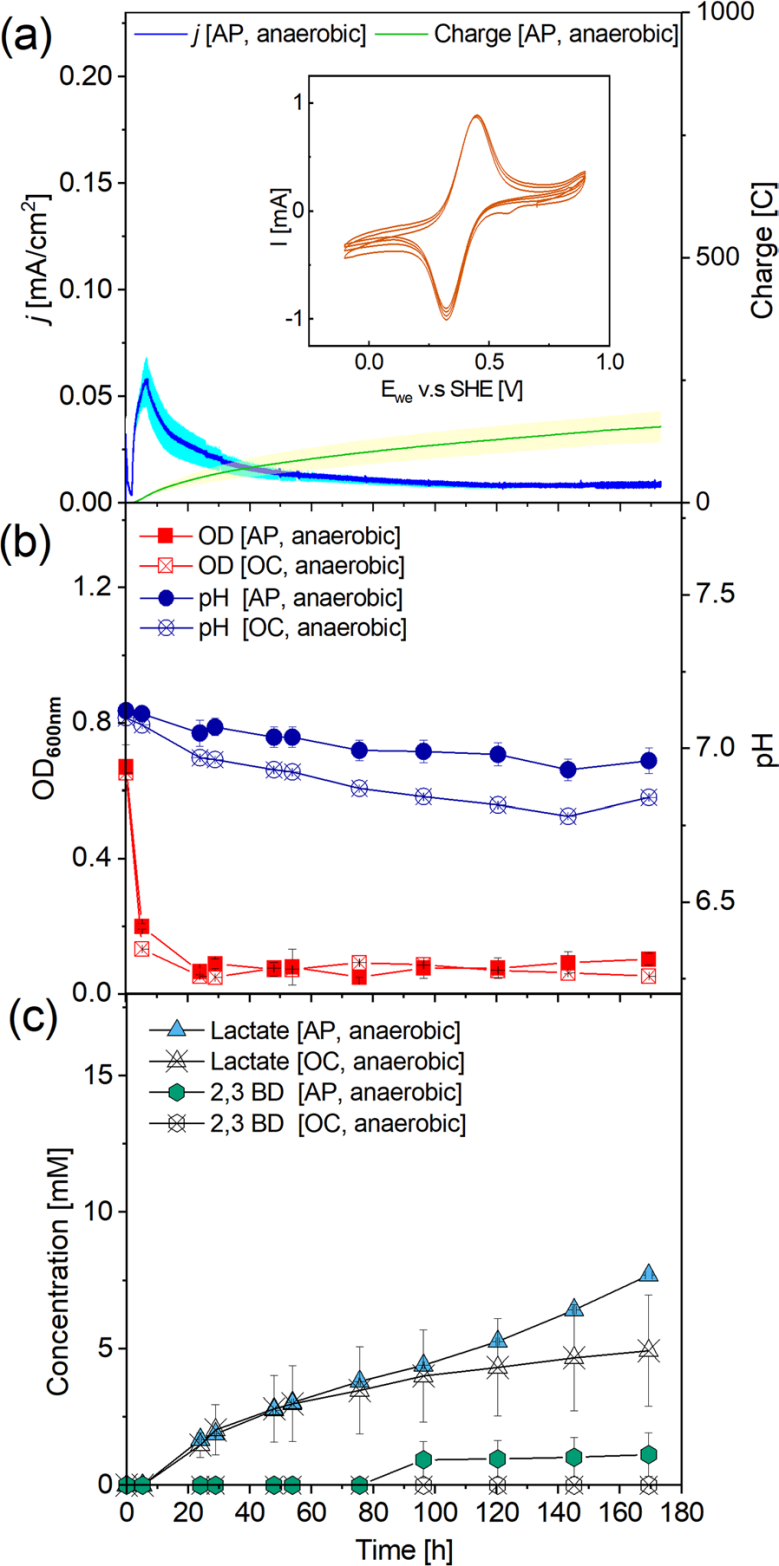
*B. subtilis* grown in anaerobic BES with glucose as substrate and K_3_[Fe(CN)_6_] acting as a mediator. Current density and charge logged at poised anode potential 0.7 V and abiotic electrochemical characterisation of the mediator via CV using graphite felt as the working electrode **a**. Optical density and pH **b**, glucose, lactate and 2,3-butanediol concentrations **c** were measured from the reactors with applied potential [AP] and open circuit [OC]. Results are based on average data of three biological replicates and standard deviations are represented as coloured area and error bars

To investigate whether an anode can be used as the sole electron acceptor, *B. subtilis* was inoculated in BESs operated under anaerobic conditions. In addition, K_3_[Fe(CN)_6_] was tested and added as a redox mediator to enhance the microbial extracellular electron transfer to the anode. The abiotic result of the cyclic voltammetry after adding K_3_[Fe(CN)_6_] shows the oxidative and reductive peaks at 0.3 and 0.6 V, respectively, with a midpoint potential of 0.5 V (Fig. 2a). Briefly, in the biological experiments, the added 1.5 mM K_3_[Fe(CN)_6_] boosted the overall current densities in BES and showed no significant inhibition on the aerobic growth of *B. subtilis* in shake flasks (for aerobic growth with different K_3_[Fe(CN)_6_] concentrations (Additional file 1: Fig. S6)). After inoculating the BES, the current density immediately increased to 0.05 mA/cm^2^ and then gradually dropped to 0.01 mA/cm^2^ after 50 h. In total, 155.9 ± 31.6 C was transferred to the anode over 170 h. The planktonic cell density decreased from *OD*_*600*_ 0.7 to 0.2 in 2 h after the inoculation and remained stable afterwards. A minor drop in pH from 7.1 to 6.9 was observed (Fig. 2b). Glucose uptake was hindered in the anaerobic BES with less than 0.01 mmol/L/h glucose consumption rate and 80% of the added glucose was left after 170 h (Fig. 2c). Lactate and 2,3-butanediol were the only metabolites detected (Fig. 2c) with end-point concentrations of 7.7 ± 1.0 mM and 1.1 ± 0.8 mM, respectively. The open-circuit reactors showed almost identical results with only lower end-point concentrations of lactate (4.9 ± 2.0 mM) and no 2,3-butanediol detected (Fig. 2c).

### Anode-assisted respiration of *B. subtilis* under moderate aeration

**Fig. 3:**
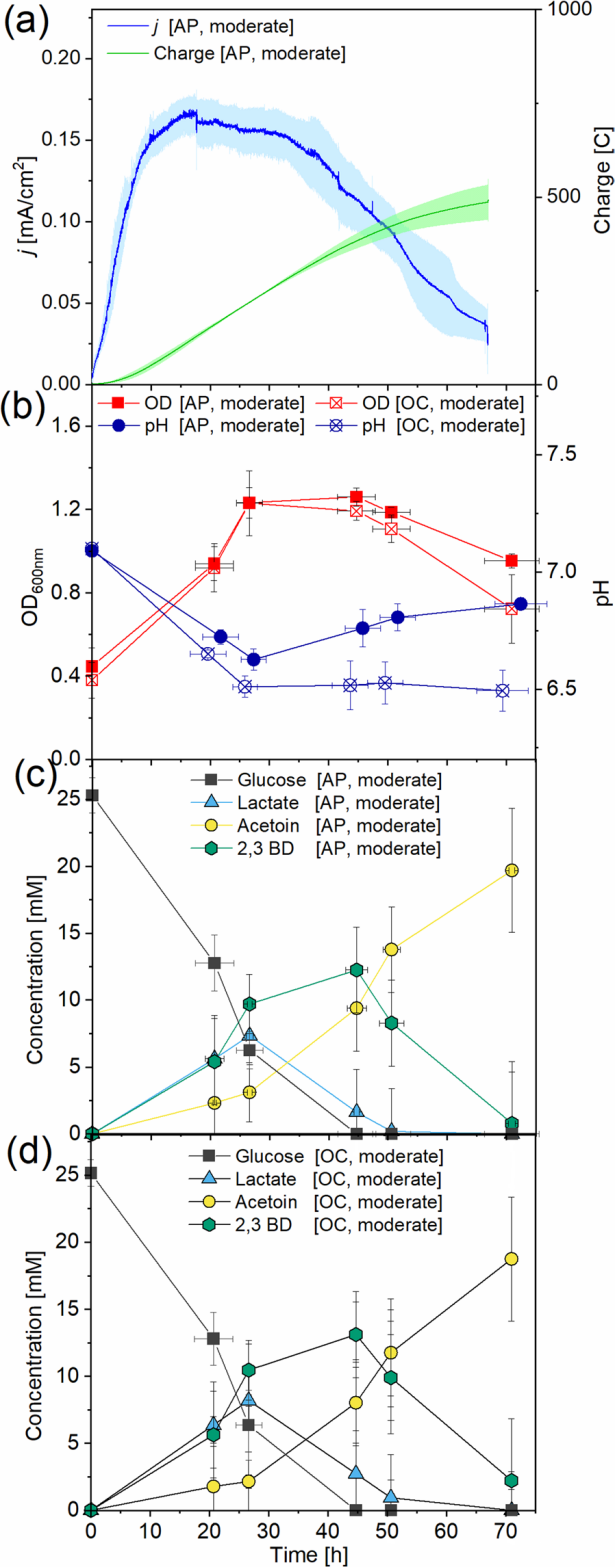
*B. subtilis* grown in BES under the moderate aeration condition with glucose as substrate and K_3_[Fe(CN)_6_] acting as a mediator. Current density and charge logged at a poised anode potential of 0.7 V **a**. Optical density and pH were measured from reactors with applied potential [AP] and open circuit [OC] **b**, and glucose and end product concentrations were measured from AP **c** and OC **d**. Concentrations for acetate (Additional File 1: Fig. S3) are shown in Additional File 1. Results are based on average data of three biological replicates and standard deviations are represented as coloured area and error bars

Based on the anaerobic serum flask results, oxygen is obligatory for *B. subtilis.* Thus, anode-assisted aerobic respiration was investigated by aerating the anolyte under a moderate aeration rate (30 mL/min). In total, three-fold more charge was transferred to the anode (491.9 ± 56.6 C) compared to the anaerobic condition with a three-fold higher current density reached (up to 0.17 mA/cm^2^) (Fig. 3a). The highest planktonic cell density (*OD*_*600*_ = 1.3) was measured after 44.7 h (Fig. 3b) under applied potential and open-circuit conditions. Similar concentrations of metabolites were detected under AP and OC (for statistical significance results, see Additional file 1: Table S2), and acetoin as the main metabolite reached an average end-point concentration of 19.2 mM, followed by 2,3-butanediol (12.7 mM, peak concentration), lactate (7.8 mM, peak concentration) and acetate (3.2 mM, peak concentration) (Fig. 3c, Additional file 1: Fig. S3). Lactate and 2,3-butanediol concentrations decreased after 26.6 h and 44.7 h, respectively, under both conditions with average consumption rates of 0.3 and 0.4 mmol/L/h, respectively. Lactate and 2,3-butanediol as intermediate products were depleted completely at the end of the experiment. A slightly higher pH was measured in the AP (pH 6.9) compared to the OC reactors (pH 6.5). Overall, 82% carbon and redox balances were achieved, with an acetoin yield of 0.8 mol_product_/mol_glucose_ under both conditions.

### Anode-assisted respiration of *B. subtilis* under limited aeration

**Fig. 4:**
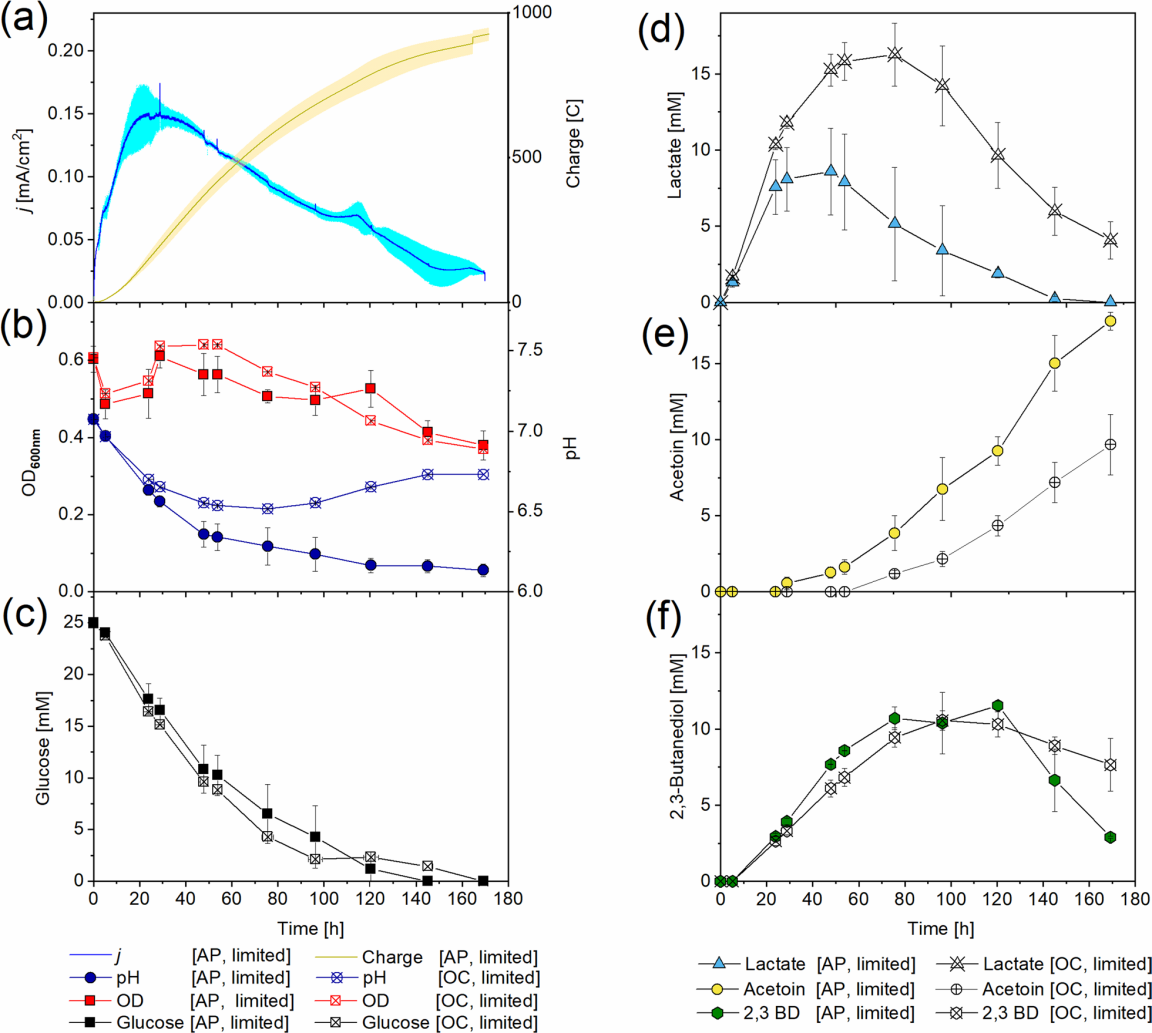
*B. subtilis* grown in BES under the limited aeration condition with glucose as substrate and K_3_[Fe(CN)_6_] acting as a mediator. Current density and charge logged at a poised anode potential of 0.7 V **a**. Optical density and pH **b**, glucose, lactate, acetoin and 2,3-butanediol concentrations **c**–**f** were measured from reactors with applied potential [AP] and open circuit [OC]. Results without the addition of K_3_[Fe(CN)_6_] (Additional file 1: Fig. S5) are shown in Additional file 1. Results are based on average data of three biological replicates and standard deviations are represented as coloured area and error bars

When less oxygen was available by decreasing the air flow rate from moderate (30 mL/min) to limited (5 mL/min), *B. subtilis* showed different characteristics of growth and product spectrum. In reactors under the poised anode potential, a current density of 0.15 mA/cm^2^ was reached after 24 h (Fig. 4a), resulting in a total charge transfer of 926.2 ± 22.7 C to the anode i.e. 1.9 times more than under the moderate aeration. A steady planktonic cell density was measured (*OD*_*600*_ = 0.6) and pH decreased over time from 7.1 to 6.1. Glucose was progressively consumed with an average rate of 0.2 mmol/L/h and depleted after 145 h (AP) and 169 h (OC) (Fig. 4c). Acetoin fermentation was dominant in AP reactors with a peak concentration of 17.8 ± 0.6 mM, as opposed to OC where the highest concentration of lactate (16.3 ± 2.1 mM) was measured. Both moderate and limited aeration led to similar amounts of 2,3-butanediol or acetate (Additional file 1: Figs. S3, S4d, e, f). Under limited aeration, lactate and 2,3-butanediol concentrations decreased after 47.8 h and 120.4 h, respectively, with three-fold slower consumption rates than under moderate conditions (0.1 mmol/L/h for lactate and 2,3-butanediol). Overall, 85% carbon and redox balances were calculated under limited aeration, with the highest acetoin yield (0.7 ± 0.02 mol_product_/mol_glucose_) found in AP reactors.

## Discussion

### The role of oxygen for *B. subtilis* growth and end product spectrum

In this study, the absence of oxygen restricted the metabolic activity of *B. subtilis* and resulted in abrupt cell lysis, although many studies have shown *B. subtilis* can grow without oxygen using alternative electron acceptors, including nitrate [13, 32, 33]. Among all the tested conditions, the highest cell density (*OD*_*600*_ = 5.1) was measured in aerobic shake flasks with an average doubling time of 1.6 h. A typical oxygen volumetric mass transfer coefficient (k_L_a) in similar aerobic shake flasks and growth conditions is ranging from 160 to 226 h^–1^ [34], nearly five times higher compared to the estimated 36 h^–1^ in H-type reactors used with moderate aeration in anode-assisted electro-fermentation in this study [35]. When nitrate or anode was used as the alternative electron acceptor by *B. subtilis*, lower glucose consumption rates (< 0.7 mM/h) and cell densities (*OD*_*600*_ < 2) compared to aerobic shake flasks (2.3 mM/h) were observed, indicating that limited energy was available with restricted biomass formation when using the alternative electron acceptors. Oxygen as the electron acceptor facilitates sugar metabolism, energy conservation and nicotinamide cofactors, e.g. nicotinamide adenine dinucleotide (NAD^+^), regeneration in many microorganisms [2]. In *B. subtilis*, reduced dissolved oxygen concentration during cultivations has also earlier shown enhanced fermentation products with 50% less biomass formed [24].

According to the results, low oxygen supply enabled *B. subtilis* to donate electrons to alternative electron acceptors. The results suggested that low concentrations of oxygen were essential for cells to activate nitrate and anode-assisted respiration. Oxygen might not only function as electron acceptor but could have also acted as the transcriptional signal chemical to enable the anaerobic pathway [35]. For example, oxygen limitation induces the transcription of anaerobic genes and regulators for nitrate respiration in *B. subtilis* [36]. For anode-assisted respiration, oxygen may have played a more indispensable role as electron acceptor, since constant supply of oxygen was needed. At the energy level, nitrate respiration releases more free energy than fermentation: ΔG^0^ = − 2870 kJ/mol_glucose_ for oxygen, − 858 kJ/mol_glucose_ for nitrate and − 218 kJ/mol_glucose_ for fermentation, respectively [2, 37, 38]. Cell growth (*OD*_600_ = 1.7) was also observed under the microaerobic condition with consumption of nitrate in contrast to fast cell lysis under anaerobic conditions in this study. However, the fundamental understanding of nitrate respiration in *B. subtilis* under the microaerobic condition remains to be further examined.

Different aeration schemes affected the end product spectrum of *B. subtilis*. In aerobic shake flasks, acetate was the main end product (15.2 mM), whereas in BES experiments with both aeration schemes one-fourth of acetate concentration was measured. In *B. subtilis*, acetate is typically formed from acetyl-CoA via a two-step reaction encoded by *pta* and *ackA* and the formation of acetate during aerobic respiration is explained by metabolic overflow, which activates fermentative pathways to allow faster ATP production per unit cell membrane area [39–41]. During the nitrate respiration, besides acetate formation, other fermentation products, including acetoin and 2,3-butanediol, also started to appear. An increase in overall fermentative activities can be expected when oxygen is not available for the cofactors’ regeneration. In *B. subtilis*, increased NADH-coupled products secretion, i.e. lactate and 2,3-butanediol (up to 16.3 mM and 12.3 mM, respectively), was observed under the oxygen-limited conditions in BESs. However, lactate was not excreted in nitrate-added serum flasks. It has been reported that under nitrate respiration fermentation is less preferred by *B. subtilis* in terms of cofactors regeneration [36] and that the presence of nitrate under oxygen-limited conditions can drastically decrease fermentative enzyme activities, including lactate dehydrogenase [42].

### Anode-assisted electro-fermentation with *B. subtilis*

Previous anodic electro-fermentation studies have demonstrated that the combination of poised anode potential (0.7 V vs. SHE) and K_3_[Fe(CN)_6_] as the redox mediator enhanced the anaerobic metabolic activities of *Pseudomonas putida* F1 and *Corynebacterium glutamicum* lysC [25, 26, 43]. Under anode respiration in this study, *B. subtilis* cells were unable to maintain metabolic activity showing incomplete glucose oxidation. In contrast to the shake flasks, anaerobic BESs had almost zero glucose consumption, no planktonic cell growth and limited end product formation. Moderate aeration resulted in at least three-fold more charge transferred to the anode compared to anaerobic conditions. However, under moderate aeration, the anode did not show significant impacts on changing the product spectrum and oxygen was likely still acting as the main electron acceptor. With moderate aeration, the anode could not function as an electron sink for cells to uninterruptedly balance the redox per se causing almost identical end product concentrations with or without poised anode potential.

When the oxygen supply was switched to limited aeration in BESs, cells were forced to seek alternative electron acceptors and an increased number of surplus electrons (926.2 ± 22.7 C, limited aeration) with enhanced acetoin formation were observed. Chen et al. [44] suggested that NAD(H)^+^ played an important role during the extracellular electron transfer of *B. subtilis*, which likely explained the different NADH-coupled metabolites showed in this study: as the total amount of electrons shuttled to the anode increased over time, cofactor levels might have been altered by the anode, in which case less NADH-coupled metabolites are needed to balance the redox state (e.g. lactate and 2,3-butanediol). In *B. subtilis,* NAD^+^ regeneration is typically mediated by cytoplasmic lactate dehydrogenase [45]. One hypothesis is that in anode-assisted systems under limited aeration, NAD^+^ was regenerated in several ways; hence, the lower lactate concentration was observed under the poised potential. However, anode did not significantly influence other fermentation steps which involved NAD^+^ regeneration, e.g. production of 2,3-butanediol from acetoin, as similar levels of 2,3-butanediol productivities were observed under limited aeration with poised potential (0.11 ± 0.02 mmol/L/h) and in open circuit (0.09 ± 0.03 mmol/L/h). On the other hand, if the anode helped to increase the cofactor levels, such as NAD^+^, the conversion of lactate to pyruvate might also have been accelerated. Pyruvate can either re-enter the TCA cycle to potentially enhance the acetoin production or accumulate in cells to trigger the acetoin excretion as a strategy for cells to maintain the intercellular pH during the stationary phase [45]. Alternatively, the applied anode potential may also change the oxidation–reduction potential (ORP) of the fermentation broth or the intracellular redox state, the principle of which is still to be discovered [47].

### Higher acetoin yield with anode-assisted electro-fermentation systems

**Fig. 5:**
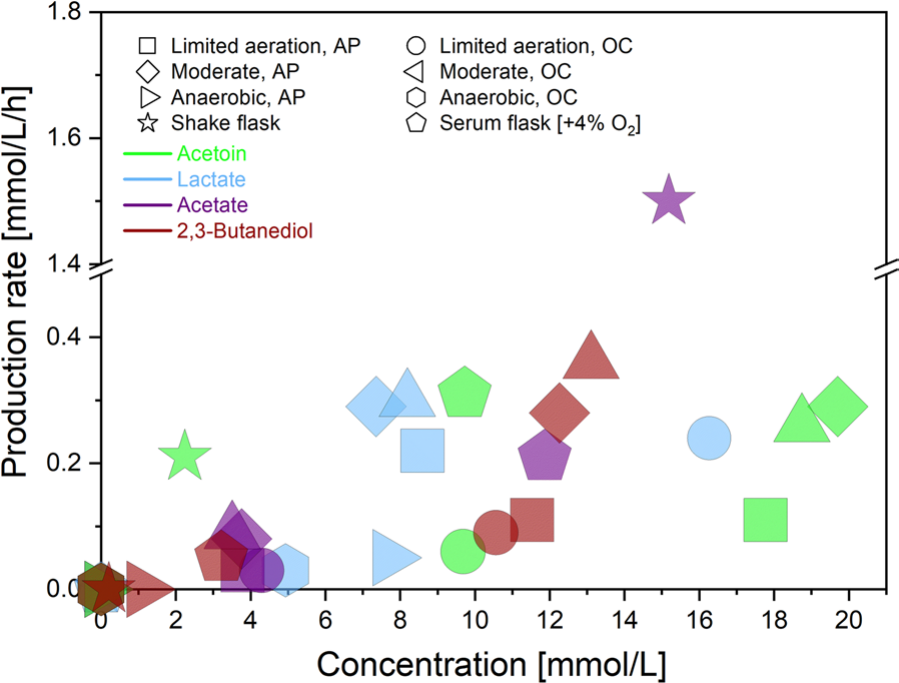
Maximum production yields (x-axis) associated with production rates (y-axis) under different conditions tested with oxygen, nitrate and anode as electron acceptors. In contrast to acetoin (neon green) and acetate (metallic violet), intermediate products 2,3-butanediol (crimson red) and lactate (light azure) concentrations declined during the experiments; therefore, only the highest production rates are shown

Adaptation of *B. subtilis* to alternative electron acceptors revealed future perspectives of using anode-assisted electro-fermentation under oxygen-limited conditions for enhanced acetoin production. First, among all tested conditions (Table 1), the high yield of acetoin (0.71–0.78 mol_product_/mol_glucose_) achieved under oxygen-limited conditions (Fig. 5) is comparable to the acetoin production of metabolically engineered *B. subtilis* strains (from 0.62 to 0.77 mol_product_/mol_glucose_ [48–50]). Although the highest production rate of acetoin (0.3 ± 0.1 mM/h) was found under nitrate respiration, a lower yield (0.3 ± 0.01 mol_product_/mol_glucose_) indicated the limitation for acetoin production under the anaerobic respiration. The results suggested that aerobic acetoin bioprocesses can be optimised with a very low air supply rate along with poised anode potential. A low aeration rate can bring financial benefits regarding operational costs and fewer foaming-related issues for industrial applications [2]. Furthermore, another industrially related strain, *P*. *putida* KT2440, was reported to improve glycolipid surfactants production under similar oxygen-limited conditions with poised anode potential [51], highlighting the potential of anodic electro-fermentation to decrease the oxygen requirements of aerobic bioprocesses.

## Conclusions

The biomass production, alternative electron acceptors utilisation and end product spectrum of the *B. subtilis* strain differed under different oxygen supplies. Both nitrate and anode-assisted respiration of *B. subtilis* occurred under oxygen-limited conditions, while fast cell lysis was observed in strictly anaerobic environments. Overall, depending on the electron acceptor the biochemical production shifted from acetate in aerobic conditions to acetate and acetoin under nitrate respiration, and further to mostly acetoin in BESs. Under limited aeration, poised anode potential at 0.7 V showed steered product spectrum towards acetoin production (17.8 ± 0.6 mM at applied potential vs. 9.7 ± 2.0 mM at open circuit), with lactate and 2,3-butanediol as intermediate products. In such anode-assisted systems, a high acetoin yield of 0.7 mol_product_/mol_glucose_ was achieved with more balanced energy distribution between biomass production and end product formation compared to systems without poised anode potential or aerobic shake flask experiments. This research highlighted the potential of anode-assisted electro-fermentation to connect an industrial aerobic microorganism with bioelectrochemical systems with reduced oxygen dependency for biochemical production.

## Supplementary Information

**Additional file 1: Fig S1. ** Schematic figure and photo of bioelectrochemical setup used in this study. **Fig S2.** Headspace gas compositions measured in microaerobic serum flasks. Results are based on the average data of three biological replicates. **Fig S3.** Acetate concentrations measured in BES systems under different cultivation conditions (see **Table 1**, **main article**). Results are based on the average data of three to four biological replicates and standard deviations are represented as error bars. **Fig S4.** *B. subtilis *grown in BES under limited aeration without adding K_3_[Fe(CN)_6_]. Ten times less current density and charge were logged at a poised anode potential of 0.7 V (vs. SHE) reactors compared to reactors amended with K_3_[Fe(CN)_6_] (a). Optical density and pH (b), glucose, lactate, acetoin and 2,3-butanediol concentrations (c, d, e, f) were measured from applied potential (AP) and open-circuit (OC) reactors. Results are based on the average data of four biological replicates and standard deviations are represented as coloured areas or error bars. **Fig S5.** *B. subtilis *grown in aerobic shake flasks with the addition of 0, 0.5, 1.5 and 5 mM K_3_[Fe(CN)_6_] in the M9 medium with 5 g/L glucose. The backscatter value corresponds to the biomass density. Each curve is based on the average data of three biological replicates and standard deviations are represented as coloured areas. **Table S1.** Main parameters of *B. subtilis *grown using different electron acceptors. n.d.: not detected; [-]: without the addition of K_3_[Fe(CN)_6_]; biomass and CO_2_ concentrations were not included for carbon and redox balances. **Table S2.** Student’s t-test results to compare the main parameters of *B. subtilis *growing in BES under three different conditions: t-tests have been performed via Microsoft Excel (data set of three (3 replicates), distribution tails = 1, two-sample unequal variance) to prove the null hypothesis (no difference between the conditions). To reject the null hypothesis a probability value (*p*-value) of lower than 0.05 has been chosen (*p *< 0.05 →value in bold). A: limited aeration, 1.5 mM K_3_[Fe(CN)_6_], AP; B: limited aeration, 1.5 mM K_3_[Fe(CN)_6_], OC; C: limited aeration, no K_3_[Fe(CN)_6_], AP; D: moderate aeration, 1.5 mM K_3_[Fe(CN)_6_], AP; E: moderate aeration, 1.5 mM K_3_[Fe(CN)_6_], OC.

## Data Availability

Not applicable.

## Abbreviations

ADP: Adenosine diphosphate
ATP: Adenosine triphosphate
AP: Applied potential
BES: Bioelectrochemical system
CV: Cyclic voltammetry
FADH_2_: Flavin adenine dinucleotide
GC: Gas chromatography
HPLC: High-performance liquid chromatography
IC: Ion chromatography
NAD^+^/NADH: Nicotinamide adenine dinucleotide (oxidised/reduced)
NADP^+^/NADPH: Nicotinamide adenine dinucleotide phosphate (oxidised/reduced)
OC: Open circuit
OD: Optical density
SHE: Standard hydrogen electrode
TCA: Trichloroacetic acid
