# Supplementary material for: Anode-assisted electro-fermentation with Bacillus subtilis under oxygen-limited conditions
Source: Biotechnol Biofuels Bioprod. 2023 Jan 10;16:6. doi: 10.1186/s13068-022-02253-4 (PMC9832610; doi:10.1186/s13068-022-02253-4)
Supplement: Supplementary file 1 — Additional file 1: Fig S1. Schematic figure and photo of bioelectrochemical setup used in this study. Fig S2. Headspace gas compositions measured in microaerobic serum flasks. Results are based on the average data of three biological replicates. Fig S3. Acetate concentrations measured in BES systems under different cultivation conditions (see Table 1, main article). Results are based on the average data of three to four biological replicates and standard deviations are represented as error bars. Fig S4. B. subtilis grown in BES under limited aeration without adding K3[Fe(CN)6]. Ten times less current density and charge were logged at a poised anode potential of 0.7 V (vs. SHE) reactors compared to reactors amended with K3[Fe(CN)6] (a). Optical density and pH (b), glucose, lactate, acetoin and 2,3-butanediol concentrations (c, d, e, f) were measured from applied potential (AP) and open-circuit (OC) reactors. Results are based on the average data of four biological replicates and standard deviations are represented as coloured areas or error bars. Fig S5. B. subtilis grown in aerobic shake flasks with the addition of 0, 0.5, 1.5 and 5 mM K3[Fe(CN)6] in the M9 medium with 5 g/L glucose. The backscatter value corresponds to the biomass density. Each curve is based on the average data of three biological replicates and standard deviations are represented as coloured areas. Table S1. Main parameters of B. subtilis grown using different electron acceptors. n.d.: not detected; [-]: without the addition of K3[Fe(CN)6]; biomass and CO2 concentrations were not included for carbon and redox balances. Table S2. Student’s t-test results to compare the main parameters of B. subtilis growing in BES under three different conditions: t-tests have been performed via Microsoft Excel (data set of three (3 replicates), distribution tails = 1, two-sample unequal variance) to prove the null hypothesis (no difference between the conditions). To reject the null hypothesis a probability value [file 13068_2022_2253_MOESM1_ESM.pdf]

Additional file

Anode-assisted electro-fermentation with *Bacillus subtilis* under oxygen-limited conditions

Yu Sun[a, *], Marika Kokko[a] and Igor Vassilev[a, *]

[a] Faculty of Engineering and Natural Sciences, Tampere University, Korkeakoulunkatu 8, 33720 Tampere, Finland

[*] Corresponding authors

E-mails: yu.sun@tuni.fi (Y. Sun), marika.kokko@tuni.fi (M. Kokko), igor.vassilev@tuni.fi (I. Vassilev)

Additional file:

**Fig. 1**	Schematic and photo of bioelectrochemical setup used in this study
**Fig. 2**	Headspace gas compositions measured in nitrate-added serum flasks
**Fig. 3**	Acetate concentrations for BES systems under different cultivation conditions
**Fig. 4**	*B. subtilis* grown in BES under limited aeration without adding $K_3[Fe(CN)_6]$
**Fig. 5**	*B. subtilis* grown in aerobic shake flasks with the addition of 0, 0.5, 1.5 and 5 mM $K_3[Fe(CN)_6]$ in the M9 medium with 5 g/L glucose
**Table 1**	Main parameters of *B. subtilis* grown using different electron acceptors
**Table 2**	Student's t-test results to compare the main parameters of *B. subtilis* growing in BES under 3 different conditions

Bioelectrochemical system and operation. Both chambers of the BES reactor were filled with 300 mL phosphate buffer solution (8.5 g/L $Na_2HPO_4 \cdot 2H_2O$, 3 g/L KH_2PO_4, 1 g/L NH_4Cl and 0.5 g/L NaCl) and autoclaved at 121 °C for 20 minutes under the pressure of 1.5 bars. The anolyte was emptied and changed to a sterile M9 medium that contained per litre: 8.5 g $Na_2HPO_4 \cdot 2H_2O$, 3 g KH_2PO_4, 1 g NH_4Cl, 0.5 g NaCl, 246 mg $MgSO_4 \cdot 7H_2O$, 147 mg $CaCl_2 \cdot 2H_2O$, 5 g glucose, and 10 mL filter-sterilized trace element solution, containing per litre: 1.5 mg EDTA, 450 mg $ZnSO_4 \cdot 7H_2O$, 30 mg $CoCl_2 \cdot 6H_2O$, 100 mg $MnCl_2 \cdot 4H_2O$, 30 mg $CuSO_4 \cdot 5H_2O$, 1.4 g $FeSO_4 \cdot 7H_2O$, 40 mg $Na_2MoO_4 \cdot 2H_2O$, 100 mg H_3BO_3, 10 mg KI by using the laminar flow cabinet. The reference electrode was sprayed with 70 % ethanol and cleaned with sterile MQ water before attaching it to the reactor through a glass tube with ceramic frit (4x50mm, Prosense, Germany).

Calculations. The total electrons transferred to the anode were calculated using a similar method described by Vassilev et al. (2019), i.e., the recorded current at each time point was multiplied by the time difference to the previous point (1 *A s* = 1 Coulomb (C)). Faraday constant (96485.3365 C/mol) was introduced to convert the *A s* value into mol electrons. Data of all time points were added to give the total mol of electrons and the reactor volume was taken into consideration during the calculations. The carbon balance (CB) was calculated for each measuring point until the substrate was consumed using the equation below:

$$the\ CB\ (\%) = {\sum_i (m_i \times n_i)_t} \Big/ {\sum_i (m_i \times n_i)_{t0}} \times 100$$

Where, m_i is the absolute concentration of compound *i* at a specific time *t*; n_i is the carbon atom number of compound i. Time t_0 denotes the point of inoculation. The redox balance was calculated based on the CB by additionally multiplying each compound with its degree of reduction plus the

mol electrons transferred to the anode. Carbon dioxide and biomass concentrations were not considered in the carbon and redox balance calculations. The yield coefficients were calculated by the slope of plots of mol product versus mol glucose consumed.

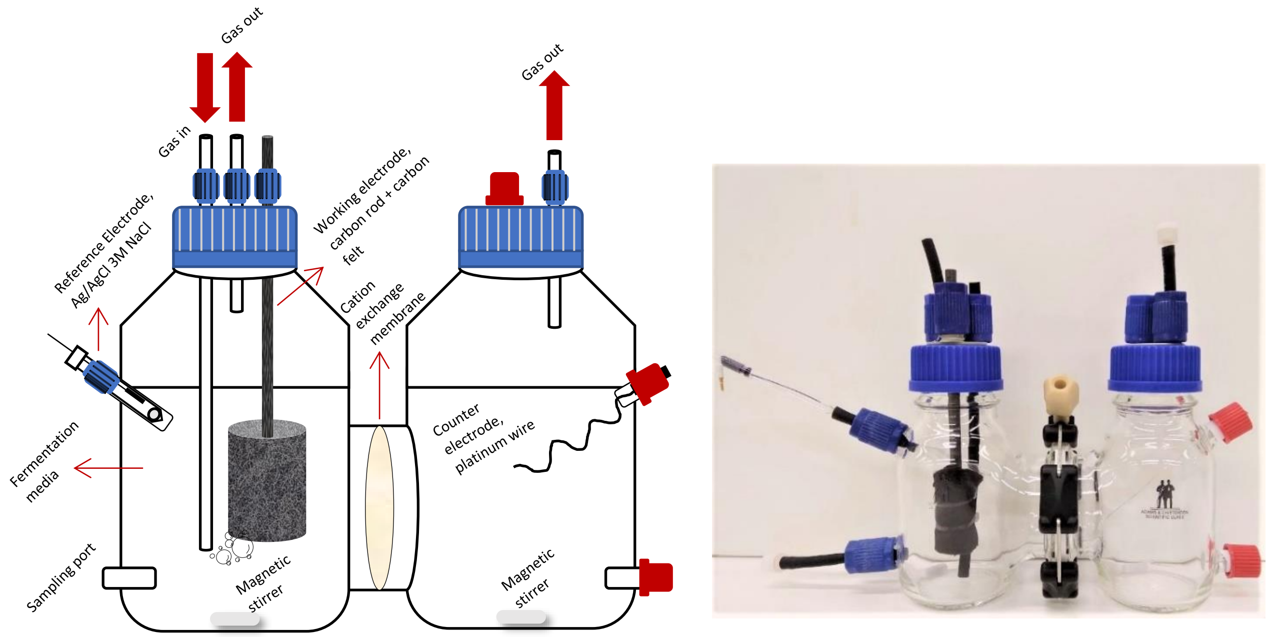

Fig. S1 Schematic figure and photo of bioelectrochemical setup used in this study.

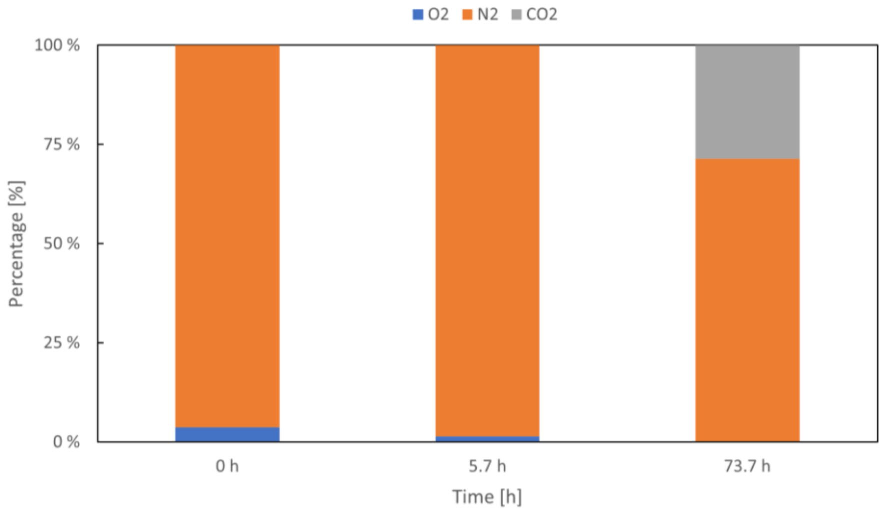

Fig. S2 Headspace gas compositions measured in microaerobic serum flasks. Results were based on the average data of three biological replicates.

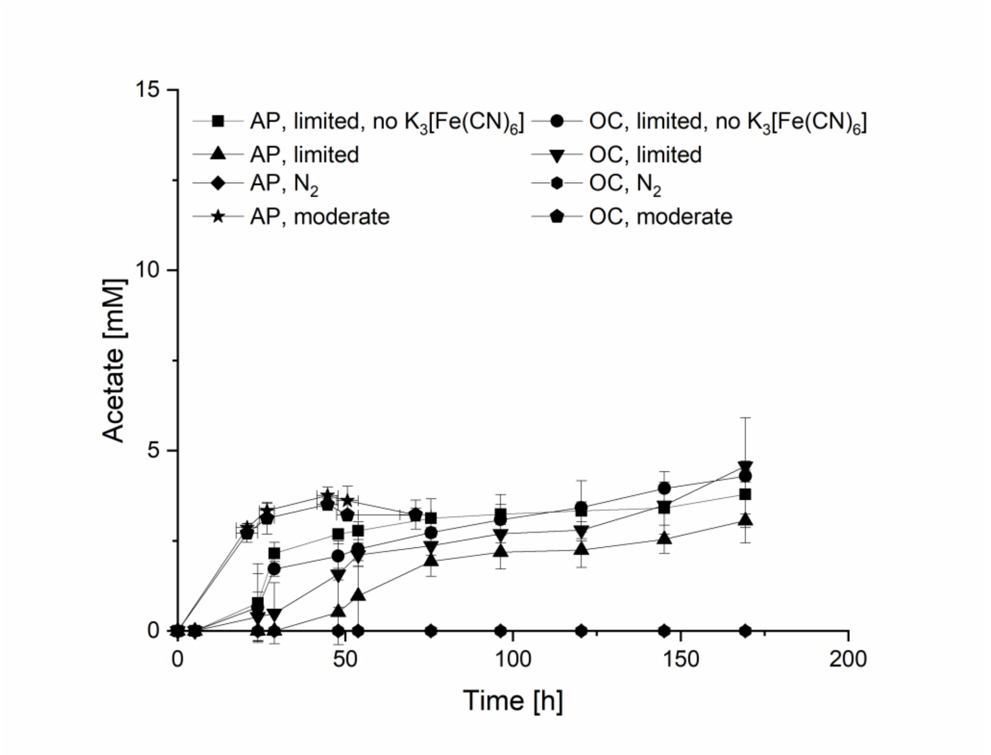

Fig. S3 Acetate concentrations measured in BES systems under different cultivation conditions (see **Table 1**, **main article**). Results were based on the average data of three to four biological replicates and standard deviations are represented as error bars.

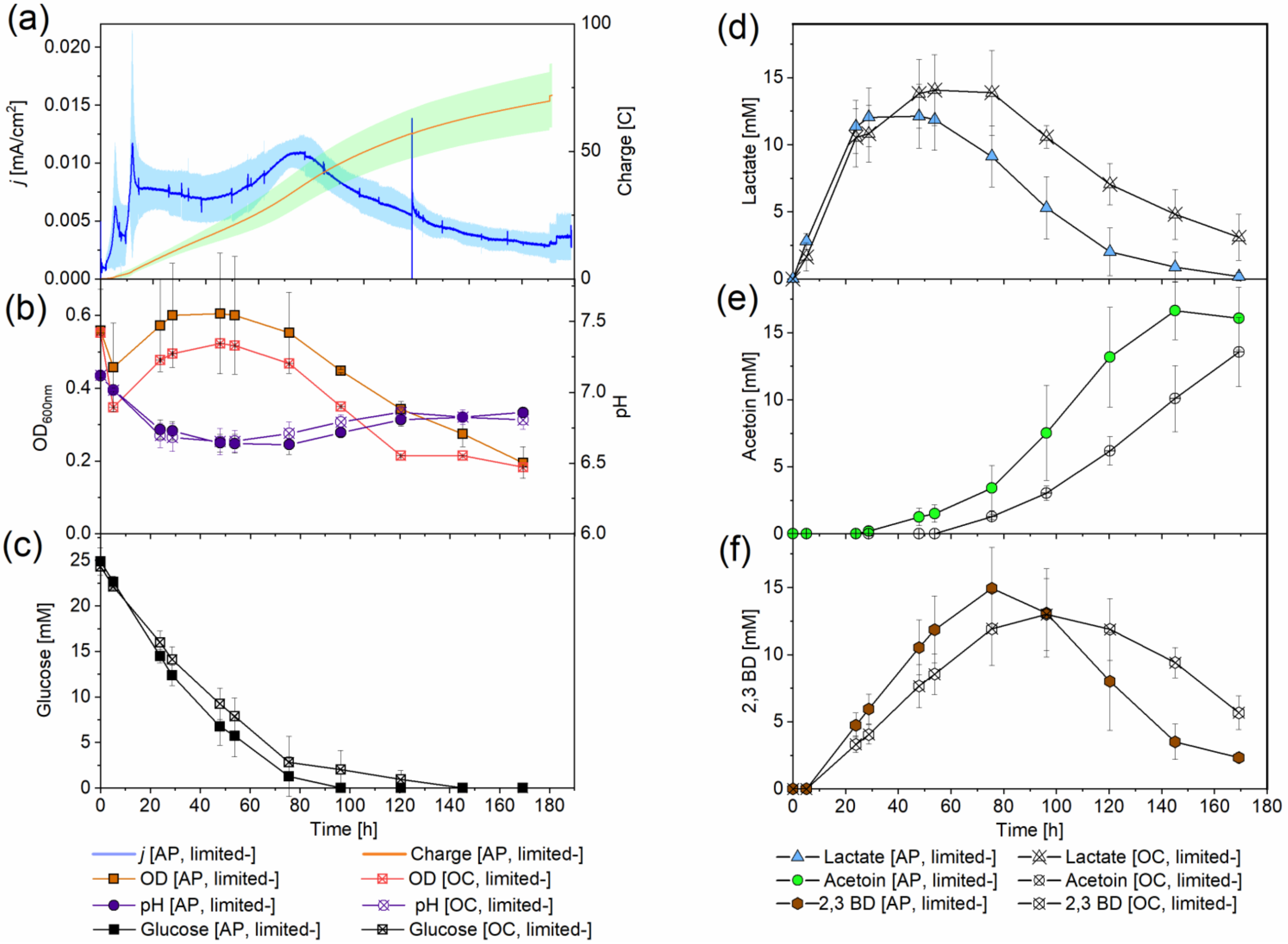

Fig. S4 *B. subtilis* grown in BES under limited aeration without adding $K_3[Fe(CN)_6]$. Ten times less current density and charge were logged at a poised anode potential of 0.7 V (vs. SHE) reactors compared to reactors amended with $K_3[Fe(CN)_6]$ (a). Optical density and pH (b), glucose, lactate, acetoin, and 2,3-butanediol concentrations (c, d, e, f) were measured from applied potential (AP) and open-circuit (OC) reactors. Results were based on the average data of four biological replicates and standard deviations are represented as coloured areas or error bars.

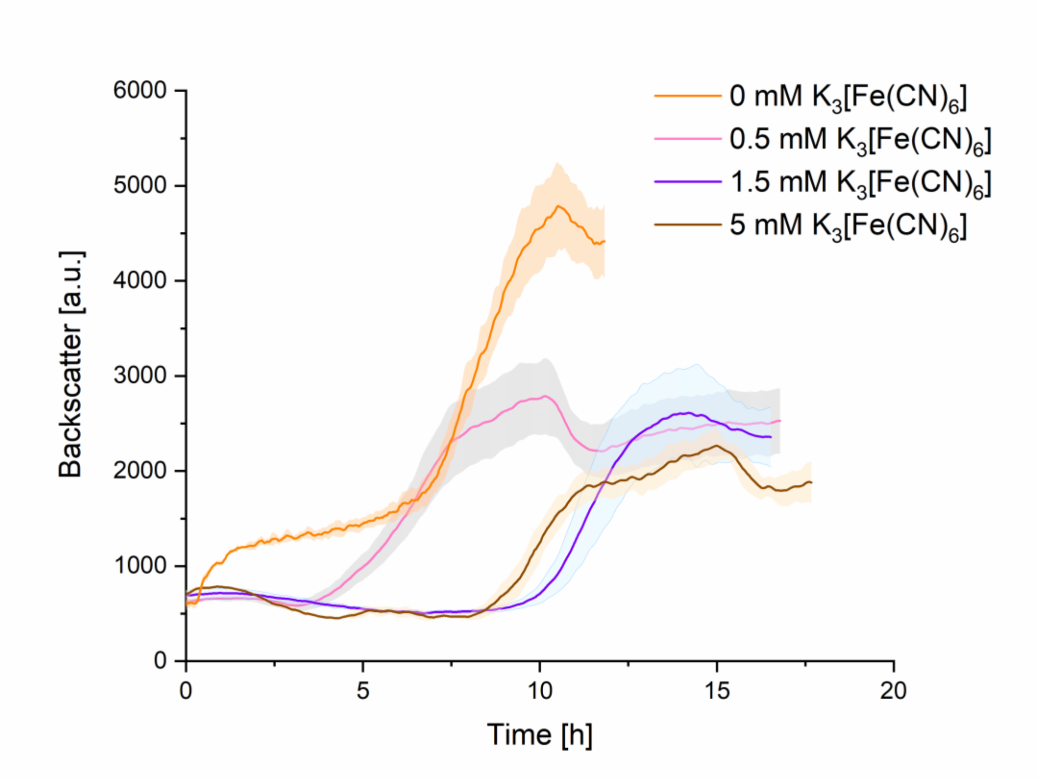

Fig. S5 *B. subtilis* grown in aerobic shake flasks with the addition of 0, 0.5, 1.5 and 5 mM $K_3[Fe(CN)_6]$ in the M9 medium with 5 g/L glucose. The backscatter value corresponds to the biomass density. Each curve was based on the average data of three biological replicates and standard deviations are represented as coloured areas.

Table S1 Main parameters of *B. subtilis* grown using different electron acceptors. n.d.: not detected; [-]: without the addition of $K_3[Fe(CN)_6]$; biomass and CO_2 concentrations were not included for carbon and redox balances.

	Shake flask	Serum flask	Serum flask [+4% O_2]	Anaerobic AP	Anaerobic, OC	Moderate AP	Moderate OC	Limited AP	Limited OC	Limited AP [-]*	Limited OC [-]*
**Carbon balance [%]**	60.70 ± 1.60	90.05 ± 3.34	65.0 ± 1.66	93.80 ± 2.25	97.48 ± 2.35	74.91 ± 1.66	76.77 ± 2.32	80.36 ± 1.89	81.20 ± 2.61	77.6 ± 1.70	80.5 ± 1.72
**Redox balance [%]**	62.21 ± 1.79	88.64 ± 1.86	69.22 ± 1.60	97.02 ± 6.23	104.66 ± 2.36	85.48 ± 2.65	90.71 ± 5.90	89.26 ± 1.98	88.32 ± 2.30	89.0 ± 1.73	89.3 ± 2.91
**Volumetric production rates [mmol/L/h]**											
**Lactate**	n.d.*	n.d.	n.d.	0.05 ± 0.01	0.03 ± 0.01	0.29 ± 0.04	0.30 ± 0.04	0.22 ± 0.04	0.24 ± 0.01	0.29 ± 0.06	0.25 ± 0.04
**Acetate**	1.50 ± 0.10	n.d.	0.21 ± 0.04	n.d.	n.d.	0.08 ± 0.01	0.09 ± 0.01	0.03 ± 0.003	0.03 ± 0.001	0.03 ± 0.01	0.02 ± 0.003
**Acetoin**	0.21± 0.01	n.d.	0.31 ± 0.08	n.d.	n.d.	0.29 ± 0.02	0.26 ± 0.02	0.11 ± 0.01	0.06 ± 0.01	0.12 ± 0.02	0.08 ± 0.02
**2,3-Butanediol**	0.02 ± 0.03	n.d.	0.05 ± 0.02	0.003 ± 0.004	n.d.	0.28 ± 0.03	0.36 ± 0.06	0.11 ± 0.02	0.09 ± 0.03	0.2 ± 0.04	0.13 ± 0.04
**Volumetric consumption rates [mmol/L/h]**											
**Glucose**	2.33 ± 0.09	n.d.	0.70 ± 0.17	0.03 ± 0.004	0.01 ± 0.01	0.58 ± 0.01	0.55 ± 0.02	0.21 ± 0.04	0.22 ± 0.05	0.31 ± 0.04	0.24 ± 0.08
**Nitrate**	n.d.	0	0.45 ± 0.10	n.d.	n.d.	n.d.	n.d.	n.d.	n.d.	n.d.	n.d.
**Lactate**	n.d.	n.d.	n.d.	0	0	0.26 ± 0.06	0.29 ± 0.09	0.07 ± 0.04	0.13 ± 0.03	0.12 ± 0.03	0.10 ± 0.05
**2,3-Butanediol**	n.d.	n.d.	0	0	0	0.44 ± 0.07	0.38 ± 0.10	0.17 ± 0.02	0.06 ± 0.02	0.15 ± 0.02	0.12 ± 0.03
**Yields [$mol_{product}$ / $mol_{glucose}$]**											
**Lactate**	n.d.	n.d.	n.d.	1.36 ± 0.34	0.28 ± 2.67	0	0	0	0.16 ± 0.05	0.01 ± 0.01	0.13 ± 0.07
**Acetate**	0.65 ± 0.02	n.d.	0.43 ± 0.02	n.d.	n.d.	0.13 ± 0.01	0.13 ± 0.02	0.15 ± 0.04	0.17 ± 0.01	0.19 ± 0.06	0.13 ± 0.03
**Acetoin**	0.1 ± 0.01	n.d.	0.32 ± 0.01	n.d.	n.d.	0.78 ± 0.04	0.75 ± 0.04	0.71 ± 0.02	0.39 ± 0.08	0.73 ± 0.04	0.56 ± 0.10
**2,3-Butanediol**	n.d.	n.d.	0.12 ± 0.02	0.10 ± 0.10	0	0.03 ± 0.02	0.09 ± 0.03	0.12 ± 0.002	0.31 ± 0.07	0.10 ± 0.02	0.23 ± 0.05

Table S2 Student's t-test results to compare the main parameters of *B. subtilis* growing in BES under 3 different conditions: t-tests have been performed via Microsoft Excel (data set of 3 (3 replicates), distribution tails = 1, two-sample unequal variance) to prove the null hypothesis (no difference between the conditions). To reject the null hypothesis a probability value (*p*-value) of lower than 0.05 has been chosen ($p < 0.05$ → value in bold). A: limited aeration, 1.5 mM $K_3[Fe(CN)_6]$, AP; B: limited aeration, 1.5 mM $K_3[Fe(CN)_6]$, OC; C: limited aeration, no $K_3[Fe(CN)_6]$, AP; D: moderate aeration, 1.5 mM $K_3[Fe(CN)_6]$, AP; E: moderate aeration, 1.5 mM $K_3[Fe(CN)_6]$, OC.

		A*<->B*	A<->C*	A<->D*	D<->E*
**Production rate [mmol/L/h]**	Lactate	0.242990722	0.080942992	0.088826462	0.367843657
	Acetate	0.33677822	0.463867591	**0.000278804**	0.317483796
	Acetoin	**0.005888725**	0.226379097	**0.000859811**	0.108204766
	2,3-Butanediol	0.269433549	**0.014031509**	**0.000984212**	0.091851823
**Yield [$mol_{product}/mol_{glucose}$]**	Lactate	**0.020414271**	0.195501109	n.a.	n.a.
	Acetate	0.255502088	0.235160147	0.233403189	0.482448804
	Acetoin	**0.012477393**	0.32345382	0.05567697	0.209364145
	2,3-Butanediol	**0.028081078**	**0.090376003**	**0.016377182**	**0.039384115**
**Consumption rate [mmol/L/h]**	Glucose	0.43162878	**0.020414486**	**0.001348747**	**0.04912412**
	Lactate	0.111922721	0.128928284	**0.014415655**	0.364155446
	2,3-Butanediol	**0.003954687**	0.184453584	**0.011837859**	0.26092092
